# A comparison of breast cancer incidence and cancer stages before and after the introduction of the Austrian national breast cancer screening program in the federal state of Salzburg: Real-world data from the Tumor Registry Salzburg

**DOI:** 10.1007/s00508-025-02508-8

**Published:** 2025-04-01

**Authors:** Simon Peter Gampenrieder, Marc Vaisband, Gabriel Rinnerthaler, Lukas Weiss, Bernhard Jaud, Martin Sprenger, Richard Greil

**Affiliations:** 1 Department of Internal Medicine III with Haematology, Medical Oncology, Haemostaseology, Infectiology and Rheumatology, Oncologic Center, Salzburg Cancer Research Institute—Laboratory for Immunological and Molecular Cancer Research (SCRI-LIMCR), Paracelsus Medical University Salzburg, Müllner Hauptstraße 48, 5020 Salzburg, Austria; 2 Cancer Cluster Salzburg, Salzburg, Austria; 3 University of Bonn, Bonn, Germany; 4 Division of Oncology, Department of Internal Medicine, Medical University of Graz, Graz, Austria; 5 Institute of Social Medicine and Epidemiology, Medical University of Graz, Graz, Austria; 6 Tumor Registry Salzburg, Salzburg, Austria

**Keywords:** Mortality, Tumor stage, Diagnosis rate, Overdiagnosis, Screening effectiveness

## Abstract

**Background:**

In January 2014 a national, quality-assured breast cancer screening program was introduced in Austria. To investigate if the program reduced the incidence of advanced breast cancer stages, we evaluated data from the Tumor Registry Salzburg, which records all cancer cases diagnosed in the federal state of Salzburg, Austria. Secondary objectives were changes in nodal status and the influence of age and urban or rural residence on stage distribution.

**Methods:**

Female patients resident in the federal state of Salzburg with a first diagnosis of breast cancer in 2010–2022 were included. For the main objectives, patients aged 45–69 years with known tumor stages were evaluated. Age-standardized incidence rates were compared between 2010–2013 and 2016–2019 by normal approximation of Poisson rates and stage distributions by ordinal logistic regression.

**Results:**

The distribution of stages 0–IV did not differ significantly between 2010–2013 and 2016–2019 (*P* = 0.380). The percentage of stage IV breast cancer decreased numerically from 9.4–4.5% (*P* = 0.141). No statistically significant differences between early stages (0–I), advanced stages (II–IV, *P* = 0. 524) and between lymph node negative and positive cases (*P* = 0.538) were detected. Neither age nor urban/rural residence had a substantial influence on tumor stage. Interestingly, the breast cancer incidence rates in Salzburg decreased nonsignificantly after the introduction of screening: annual 245.7 vs. 229.8 cases per 100,000 standard population (*P* = 0.483).

**Conclusion:**

Our findings do not support the assumption that the introduction of the Austrian breast cancer screening program significantly reduced advanced stage breast cancer in the federal state of Salzburg compared to the opportunistic screening established before.

**Supplementary Information:**

The online version of this article (10.1007/s00508-025-02508-8) contains supplementary material, which is available to authorized users.

## Introduction

In January 2014 a national quality-assured breast cancer screening program was introduced in Austria. Women between 45 and 69 years of age are invited biannually to undertake a mammography and in the case of dense breast tissue additional ultrasound with double reading of the mammograms by a second radiologist. The goal of the screening program is to detect breast cancer at an early stage and to subsequently reduce breast cancer mortality. Of the invited women in the federal state of Salzburg, a constant rate of 45% participated in the screening program between 2014 and 2021 [1]. When diagnostic mammograms are considered as well, the mammography supply rate for women aged 45–69 years is calculated as 51% [1], which is not very different from the estimated rate of 55% during the unorganized, opportunistic breast cancer screening which was performed before the start of the national screening program [2]. Irrespective of the participation rate, breast cancer screening is controversial. A systematic review from 2021, conducted on behalf of the European Commission Initiative on Breast Cancer, evaluated 10 randomized controlled trials conducted in the 1960s–1990s and including more than 600,000 women aged 38–75 years [3]. This review found a 20–23% relative and a 0.05–0.2% absolute risk reduction in breast cancer mortality over 10 years, similar to a Cochrane Review from 2013 [4]; however, for several studies included in this review the risk for bias was rated as high. When those potentially biased studies were excluded, no statistically significant reduction in breast cancer and all-cause mortality could be detected anymore [4]. In addition, screening is associated with a substantial risk of overdiagnosis and false positive results [5, 6], leading to anxiety and unnecessary interventions [7]. Furthermore, several population-based studies showed that mammography screening did not reduce the incidence of advanced tumor stages or only to a small extent [8–19] and had no detectable effect on breast cancer mortality [20–23].

A successful screening program should not only reduce breast cancer mortality but also the incidence of advanced breast cancer stages. Therefore, we investigated the data from the Tumor Registry Salzburg (TRS), which collects the epidemiological data on all tumor types diagnosed in the federal state of Salzburg and asked the question if the introduction of the national breast screening program had an influence on the tumor stage at diagnosis.

Given that younger women have a lower risk of breast cancer than older women, the absolute benefit from mammography screening is likely to vary between different age groups [24]. Therefore, we investigated the influence of age on tumor stage before and after the introduction of the screening program. Furthermore, we investigated if the place of residence (urban vs. rural) played a role in this context. Previous data have shown that women living in rural areas tend to have higher breast cancer stages at diagnosis compared to women from urban areas, probably because they face more barriers to access mammography screening and further diagnostic procedures [25].

## Patients, material and methods

### Patients

The analysis was based on data from breast cancer patients included in the TRS. Since 1983 all cancer cases diagnosed in the federal state of Salzburg must be registered in the TRS, ensuring a high level of completeness. The dataset includes information on age, gender, place of residence, date of diagnosis, tumor grade, histology, receptor status, sidedness and TNM stage.

All female patients diagnosed with invasive breast cancer or ductal carcinoma in situ (DCIS) between 1 January 2010 and 31 December 2022 and resident in the federal state of Salzburg at the time of diagnosis were included in this analysis. Patients who were recorded more than once in the registry were analyzed only at the first diagnosis and were excluded if the first diagnosis was documented before 01 January 2010. For the primary and secondary objectives of this study, only patients aged 45–69 years with known tumor stage at diagnosis (pathological stage in cases of primary surgery and clinical stage in cases of neoadjuvant or palliative systemic treatment) were included.

### Objectives

The primary objective was to evaluate if the introduction of the national breast cancer screening at the federal state of Salzburg in January 2014 had a substantial influence on the anatomical tumor stage at diagnosis, defined according to the 8th edition of the American Joint Committee on Cancer Staging Manual (AJCC 2017) [26]. In a first step every tumor stage (0 = DCIS I, II, III, IV) was investigated separately. In a second step, stages 0 and I (precursor lesions and invasive tumor ≤ 2 cm) as well as stages II–IV (advanced stages) were combined. A secondary objective was the comparison of the nodal status (negative vs. positive). For this special evaluation, patients with de novo metastatic breast cancer were excluded as nodal status was not sufficiently documented in the registry in most of the cases. Further secondary objectives were the influence of age at diagnosis (in three categories: 45–52 years, 53–60 years and 61–69 years) and place of residence (urban or rural) on tumor stage and nodal status. The place of residence was categorized according to the Urban-Rural-Typologies of STATISTICS AUSTRIA [27]. In exploratory analyses, age groups outside the core target group for screening (< 45 years and ≥ 70 years) and the differences in breast cancer subtypes, i.e. hormone receptor (HR) positive (+)/human epidermal growth factor 2 (HER2) negative (−), HR+/HER2+, HR−/HER2+ and HR−/HER2− were investigated. As an additional exploratory analysis, imputation of the M‑stage was performed in patients with missing data regarding the presence of distant metastases (Mx): if T‑stage and N‑stage were available and resulted in AJCC stage I or II, M‑stage was classified as M0.

### Time periods

In this study four different time periods were defined:

1. 2010–2013: opportunistic mammography screening only (baseline period);
2. 2014–2015: introduction of the national breast cancer screening;
3. 2016–2019: established national breast cancer screening (reference period);
4. 2020–2022: potential influence by the COVID-19 pandemic.

For all objectives of this study only baseline and reference period were compared.

### Ethics

The study was approved by the ethics committee of the federal state of Salzburg (IRB Nr. 1170/2023) and was performed in accordance with the ethical standards laid down in the 1975 Declaration of Helsinki, revised in 2008.

### Statistics

Incidence rates per year per 100,000 patients were computed and age-standardized to the European standard population 2013 [28] for each observational period. To examine the incidences, we considered a Poisson model with a normal approximation following [29] yielding both point estimates and confidence intervals of the rate parameter (see appendix 1 in the supplement for mathematical details).

Moreover, ordinal and binary logistic regression [30, 31], as implemented in the statsmodels [32] package for Python, were used to investigate the influence of time period, age, place of residence and breast cancer subtypes on tumor stage and nodal status at diagnosis. As an endogenous variable, tumor stage or nodal status at diagnosis was chosen, while the exogenous variables were time period, age, place of residence (urban vs. rural) and an interaction term between age and time period to account for a potential change in the age distribution between the different time periods.

For a visualization of the case incidence rate varying over time and not just aggregated between observational periods, we furthermore used a nonparametric Bayesian inference framework by Gugushvili et al. [33], which models case incidence as a Poisson point process where intensity measure is determined by a piecewise constant density function. The result is a Bayesian estimate of the instantaneous incidence rate and is shown in Figure S1 of the supplement.

## Results

**Fig. 1:**
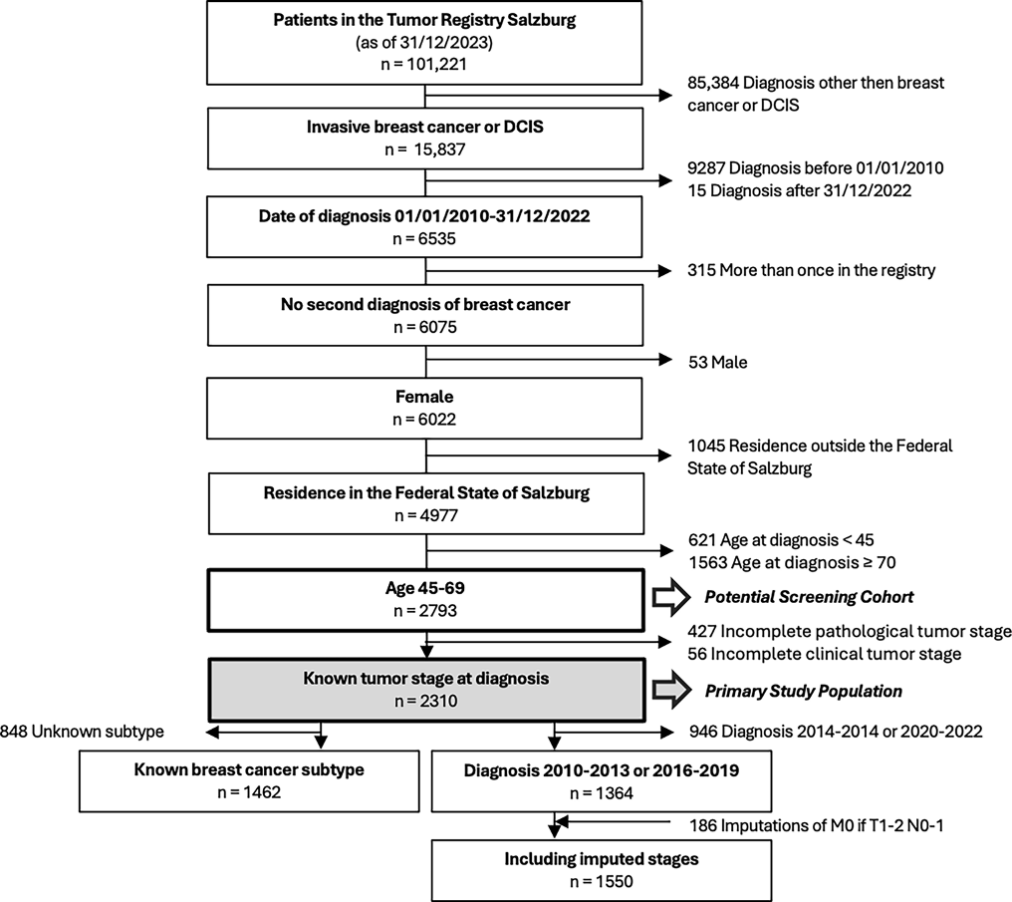
Consort diagram visualizing the patient selection process of the analysis. *DCIS* ductal carcinoma in situ

Out of approximately 95,000 women aged 45–69 years living in Salzburg between 2010 and 2022, 2793 breast cancer cases, including 292 cases of DCIS (stage 0), were registered in the Tumor Registry Salzburg (potential screening cohort; Fig. 1). This corresponds to an average age-standardized incidence rate of annually 228.5 cases per 100,000 standard population (95% CI 198.6–260.8).

**Fig. 2:**
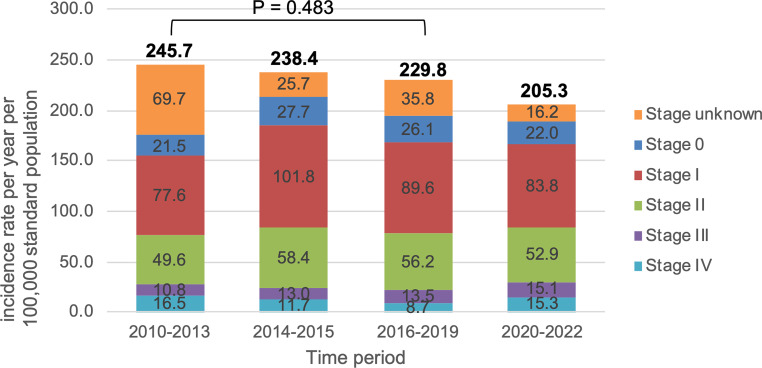
Age-standardized incidence rate per year per 100,000 standard population in the potential screening cohort aged 45–69 years (*n* = 2793). Stage according to AJCC version 8 [26]

Since the introduction of the national breast cancer screening program, the average age-standardized incidence rate in this cohort continuously decreased over time from 245.7 (95% CI 213.8–280.4) per 100,000 standard population in the 2010–2013 period to 205.3 (95% CI 177.5–235.6) in the 2020–2022 period. The difference between 2010–2013 and 2016–2019, however, was not statistically significant (*P* = 0.483; Fig. 2). When the incidence rates were evaluated over time without categorization in time periods, a short incidence peak was recorded in the first half year of 2015 (Figure S1 in the supplement).

### Primary objective

For the primary analysis 483 patients (17.3%) without adequately documented initial tumor stage (Fig. 2) had to be excluded, resulting in 2310 breast cancer patients with known tumor stage (primary study population; Fig. 1). Based on an average female population of 95,104 between 45 and 69 years in the federal state of Salzburg, the average age-standardized incidence rate was 189.1 (95% CI 162.0–218.8 per year per 100,000 standard population). The main patient and tumor characteristics of the primary study population are provided in Table 1.

**Table 1:** Patient and tumor characteristics of the primary study population

	All patients (*n* = 2310)		2010–2013 (*n* = 622)		2016–2019 (*n* = 742)	
Median age at diagnosis (years, range)	58	(45–69)	–	–	–	–
Time period of diagnosis 2010–2013	622	26.9%	622	100.0%	–	–
Time period of diagnosis 2014–2015	393	17.0%	–	–	–	–
Time period of diagnosis 2016–2019	742	32.1%	–	–	742	100.0%
Time period of diagnosis 2020–2022	553	23.9%	–	–	–	–
Stage 0 (ductal carcinoma in situ - DCIS)	292	12.6%	76	12.2%	100	13.5%
Stage I	1048	45.4%	274	44.1%	342	46.1%
Stage II	655	28.4%	177	28.5%	215	29.0%
Stage III	157	6.8%	38	6.1%	52	7.0%
Stage IV	158	6.8%	57	9.2%	33	4.4%
Nodal status positive (without stage IV)	546	23.6%	161	25.9%	165	22.2%
Nodal status negative (without stage IV)	1604	69.4%	404	65.0%	543	73.2%
Nodal status unknown	2	0.1%	0	0.0%	1	0.1%
Grade 1	400	17.3%	76	12.2%	158	21.3%
Grade 2	1192	51.6%	302	48.6%	380	51.2%
Grade 3	514	22.3%	107	17.2%	185	24.9%
Grade unknown	204	8.8%	137	22.0%	19	2.6%
Left sided	1142	49.4%	274	44.1%	368	49.6%
Right sided	1065	46.1%	279	44.9%	364	49.1%
Bilateral	1	0.0%	0	0.0%	0	0.0%
Side unknown	102	4.4%	69	11.1%	10	1.3%
Urban	1085	47.0%	282	45.3%	356	48.0%
Rural	1225	53.0%	340	54.7%	386	52.0%

The statistical analysis yielded overall results showing that the difference in incidence rates between the observation periods, if present at all, was below the precision offered by the data. Largely overlapping confidence intervals, and thus nonsignificant test results, were found for the incidence comparison of overall cases in both the potential screening cohort (2010–2013 point estimate: 245.7, 95% CI 213.0–278.4 vs. 2016–2019 point estimate: 229.8, 95% CI 199.4–260.1; *p* = 0.483) and the primary study population (176.0, 95% CI 148.3–203.7 vs. 193.9, 95% CI 166.0–221.9; *p* = 0.371). Similar results were observed for the incidence rates of the individual stages 0, I, II, III and IV. For a detailed report of the estimation results, see Table 2**.**

**Table 2:** Age-standardized incidence rate per year per 100,000 standard population according to stage in the primary study population

*n* = 2310	2010–2022	95% CI	**2010–2013**	95% CI	2014–2015	95% CI	**2016–2019**	95% CI	2020–2022	95% CI	p*
All stages*	189.1	162.0–218.8	**176.0**	149.3–206.0	212.7	183.9–244.7	**193.9**	167.0–224.0	189.1	162.4–218.2	0.371
Stage 0	23.8	14.6–35.4	**21.5**	12.9–33.6	27.7	18.1–40.6	**26.1**	16.8–38.5	22.0	13.4–33.1	0.525
Stage I	86.0	68.1–106.9	**77.6**	60.3–98.4	101.8	82.2–124.8	**89.6**	71.5–110.7	83.8	66.4–103.9	0.376
Stage II	53.4	39.3–69.9	**49.6**	35.8–66.2	58.4	43.8–76.3	**56.2**	41.9–73.1	52.9	39.3–69.3	0.539
Stage III	12.7	6.5–22.1	**10.8**	5.0–20.2	13.0	6.7–22.8	**13.5**	7.2–23.1	15.1	8.0–24.8	0.602
Stage IV	13.0	6.6–22.5	**16.5**	8.9–27.2	11.7	5.8–21.1	**8.7**	3.6–16.6	15.3	8.4–24.8	0.141
Stages 0–I	109.9	89.6–133.2	**99.1**	79.4–122.2	129.5	107.3–155.0	**115.6**	95.0–139.3	105.8	86.3–128.3	0.280
Stages II–IV	79.2	62.1–99.2	**76.9**	59.7–97.5	83.2	65.6–104.0	**78.3**	61.6–98.2	83.4	65.9–103.3	0.912
N0	131.4	108.8–156.4	**114.1**	92.9–138.7	156.6	132.0–184.4	**142.0**	118.8–167.7	126.2	104.8–150.7	0.095
*N* +	44.4	32.0–4.8	**45.4**	32.2–61.5	43.8	31.4–31.4	**43.0**	30.6–30.6	47.6	34.8–63.5	0.807
Nodal status unknown	0.2	0.0–1.39	**0.0**	0.0–0.0	0.5	0.0–5.1	**0.3**	−0.82–1.39	0.0	0.0–0.0	0.617

*Stages according to AJCC version 8

**Fig. 3:**
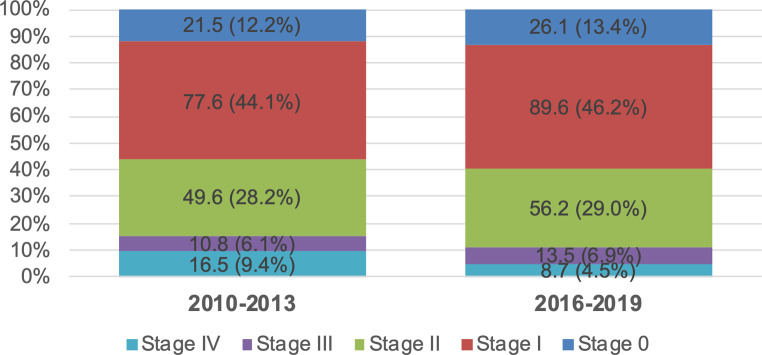
Age-standardized incidence rate per year per 100,000 standard population for the stages 0–IV and corresponding percentages in the primary study population (age 45–69 years; *n* = 2310). Stages according to AJCC version 8

This was consistent with the accompanying analysis on the distribution of stages after excluding the unknown tumor stages (where the proportion was not evenly distributed between the different time periods: 28.6% 2010–2013 vs. 15.8% 2016–2019; see Fig. 2, as well as Figure S2 in the supplement for the pattern of the age-standardized incidence rates after exclusion of unknown stages). After the introduction of the screening program, the distribution of stages 0–III remained very similar with only a small increase of all 4 stages from 2010–2013 to 2016–2019 (Fig. 3 and Table S1 in the supplement). The proportion of stage IV (de novo metastatic) breast cancer accordingly decreased from 9.4% to 4.5%; however, these differences were not of a magnitude which could be clearly differentiated from noise in the ordinal logistic regression analysis, (model coefficient : −0.155, 95% CI −0.50 to +0.19; *p* = 0.380; cf. Table 3). The results did not change when early stages (stage 0 and I) and advanced stages (II–IV) were combined (log-OR : −0.12, 95% CI −0.50 to +0.26; *p* = 0.524; see Figure S3, Figure S4 and Table S2 in the supplement).

**Table 3:** Ordinal logistic regression for stage 0–IV in the primary study population with diagnosis 2010–2013 and 2016–2019

*n* = 1364	Coefficient	95% CI	Standard error	z	p
**Time period**					
2010–2013* vs. 2016–2019	−0.155	−0.499 to 0.190	0.176	−0.879	0.380
**Age (years)**					
45–52* vs. 53–60	0.187	−0.189 to 0.563	0.192	0.973	0.330
45–52* vs. 61–69	< 0.001	−0.342 to 0.343	0.175	0.000	1.000
Interaction age (years)/time period**					
45–52* vs. 53–60 2010–2013* vs. 2016–2019	−0.138	−0.645 to 0.370	0.259	−0.533	0.594
45–52* vs. 61–69 2010–2013* vs. 2016–2019	0.040	−0.426 to 0.506	0.238	0.167	0.867
**Residence**					
Rural* vs. urban	−0.044	−0.240 to 0.153	0.100	−0.435	0.663

*Reference

**Included to correct for a potential change in age distribution between the different time periods

Stages according to AJCC version 8

### Secondary objectives

**Fig. 4:**
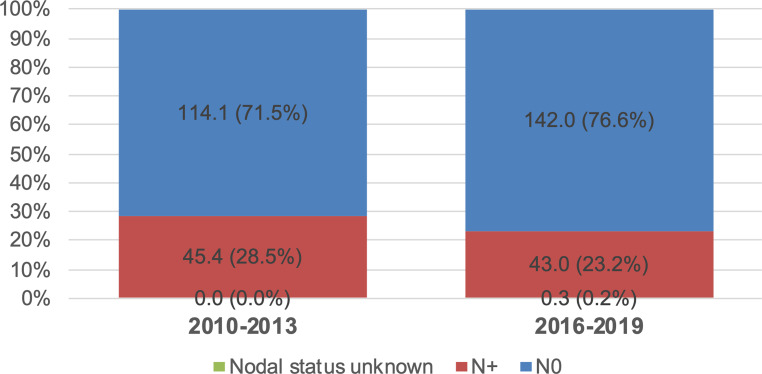
Age-standardized incidence rate per year per 100,000 standard population for node-negative vs. node-positive tumors and corresponding percentages (without stage IV) in the primary study population

When comparing node-negative with node-positive stages, a numerical decrease of node-positive tumors was observed from 2010–2013 to 2016–2019 (28.5% vs. 23.2%); however, this difference again was not statistically distinguishable from noise in the multivariable logistic regression analysis (log-OR : −0.136, 95% CI −0.57 to +0.30; *p* = 0.538; see Fig. 4, Figure S5 and Table S3 in the supplement). Furthermore, the potential influence of age and place of residence on tumor stage or nodal status at diagnosis was investigated by including these factors in the logistic regression analysis; however, in both cases, neither age nor residence had a notable effect (see Table 3).

### Exploratory outcomes

After imputation of 186 M0 values for unknown M stages (Mx) if T‑stage and N‑stage were available and resulted in AJCC stage I or II (T1–2 N0–1), the results did not change in any notable fashion (Table S4). Unfortunately, information on breast cancer subtype (HR+/HER2−; HER2+; triple negative) was unavailable for too many patients from the 2010–2013 time period (74% missing values) to allow any conclusions about different effects of breast cancer screening on different breast cancer subtypes (Figure S6 in the supplement).

## Discussion

According to the World Health Organization (WHO), the purpose of cancer screening is to detect precursor lesions or early stages in asymptomatic individuals in order to enable early treatment with better outcome and ultimately to reduce mortality and morbidity [34]. An efficient breast cancer screening program consequently should lead to an increasing incidence of early stages and correspondingly to a decreasing incidence of advanced stages of breast cancer. In several population-based trials; however, this effect was not observed [8–19]. Additionally, the prospectively shown positive effect of mammography screening on breast cancer mortality in the 1960s–1990s is thought to be “diluted” by significantly more efficient treatment options improving the prognosis of breast cancer also in advanced tumor stages [9, 35]. A recent simulation study from the USA suggested that the reduction of breast cancer mortality by 58% in the USA from 1975 to 2019 is mainly driven by improved treatments for stages I–III breast cancer (47%), while screening contributed to a much lower extent (25%) [35]. On the other side, radiology applied to the breast has advanced as well to more modern applications such as contrast-enhanced mammography (CEM), artificial intelligence, and radiomics [36], thus potentially leading to a stage shift effect with higher numbers of advanced and lower numbers of localized stages just by technological improvement.

Despite this controversy, in 2014 a national quality-assured breast cancer screening program was introduced in Austria, much later than in other European countries (e.g., Sweden 1986, United Kingdom 1989, Germany 2002) [37]. To improve the performance of the program, a supplementary breast ultrasound can be offered to women immediately after mammography at the radiologist’s discretion [38]. In order to indirectly evaluate the effectiveness of the program, we investigated the data from the Tumor Registry Salzburg to evaluate whether it reflected the anticipated changes in the patient population outlined above. We focused on disease stage at diagnosis as, in contrast to mortality, this parameter is not influenced by treatment, allowing a more direct view on screening effectiveness.

Unexpectedly, we did not find an increase in the overall incidence of invasive breast cancer and DCIS after the introduction of systematic screening. This stands in contrast to many observational trials (e.g. [9, 10]), and is most probably caused by a similar mammography supply rate during the organized national screening program (about 51% in Salzburg) [1] and the opportunistic screening before 2014 (estimated about 55%) [2]. We did observe a slight nominal shift in the empirical stage distributions, with a lower proportion (as well as incidence) of stage IV tumors; however, this could not be distinguished statistically from random fluctuations, owing to the high variance in the statistics of small numbers. Accordingly, the statistical tests performed failed to reject the hypothesis that the screening program introduction had no discernible effect. Demonstrating the absence of an impact by the introduction of the program is epistemically difficult. Our results, however, provide an upper bound on the size of any additional screening effect. This is consistent not only with the well-documented challenges of large-scale centralized screening programs but also the estimates suggesting that the rate of screening itself may not have changed much.

In a similar study, also conducted in Austria but in the federal state of Tyrol, participation in the mammography screening program was associated with a 28% risk reduction for tumors ≥ stage II and a 73% risk reduction for stage IV tumors [39]. This may appear contradictory to our results; however, substantial differences to our investigation exist both in terms of the program studied and the methodology of the investigation. Mammography screening in Tyrol started earlier (2008), included women aged 40–69 years, and invited women aged 40–59 years annually instead of biannually. Moreover, Oberaigner et al. classified patients as “exposed” or “unexposed to screening”, irrespective of the reason for breast cancer diagnosis (screening detected vs. not). As such, their results support the benefits of screening itself while our focus lies on the impact of the organized screening program.

Whether or not the trend toward lower rates of de novo metastatic breast cancer, without reduction of stages II-III tumors, can be attributed to screening cannot be answered using our dataset.

When comparing node-negative with node-positive disease (after exclusion of stage IV), more pronounced nominal differences between the two time periods were observed: while the proportion of node-negative tumors increased from 72% to 77%, node-positive tumors decreased from 23% to 29%; however, again, these effects of were within the magnitude of expected random fluctuations and thus yielded no substantial statistical evidence in favor of a benefit of the screening program.

Furthermore, we investigated if the place of residence was associated with tumor stage at diagnosis, because women living in rural areas could experience more barriers to access mammography screening than women in urban areas. Several previous datasets suggest that patients from rural areas are diagnosed at later breast cancer stages compared to women from urban areas [25]. Our results, however, did not suggest any influence of rural-urban residence or, in fact, different age categories on tumor stage at diagnosis.

One major limitation of this study is the relatively high percentage of unknown tumor stages (17%) and the uneven distribution over the different time periods, thereby hampering the comparison of incidence rates. We tried to overcome this by comparing relative distributions of tumor stages through logistic regression, alongside the incidence rate analysis. Furthermore, we performed exploratory imputation of missing information on metastatic status in otherwise early stage tumors, which did not present any different results. A potential limitation is, in addition, the fact that patients living in Salzburg could be diagnosed outside the federal state, thereby hindering the inclusion in the registry. Because of the centralized structure of the healthcare system in Salzburg, the number of affected patients is however thought to be very low.

Our observations should not be carelessly extrapolated to other countries, first and foremost due to the extent of the pre-existing opportunistic screening in Austria, a similar program in populations with less access to screening may well yield very different results. Other limitations are the lack of information on screening exposure for the patients in the registry as well as the relatively small source population of approximately 95,000 women aged 45–69 years. This reduces the precision of our estimates and limits the generalizability of our results. At the same time, this is a weakness that would be inherent to any attempt to study the net effect of the screening program on the whole breast cancer population in Salzburg and on the other side our analysis makes use of all available information on breast cancer cases in the population, including both screened and unscreened women.

In conclusion, we did not observe a reduction of locally advanced breast cancer stages (II and III) at diagnosis in the federal state of Salzburg after the introduction of the Austrian national breast cancer screening program compared to the opportunistic screening established before. These results encourage a critical evaluation of the whole Austrian breast cancer screening program.

## Supplementary Information

**Appendix 1:** Statistical details
