# Supplementary material for: A comparison of breast cancer incidence and cancer stages before and after the introduction of the Austrian national breast cancer screening program in the federal state of Salzburg: Real-world data from the Tumor Registry Salzburg
Source: Wien Klin Wochenschr. 2025 Apr 1;137(7-8):205–13. doi: 10.1007/s00508-025-02508-8 (PMC12006215; doi:10.1007/s00508-025-02508-8)
Supplement: Supplementary file 1 — Appendix 1: Statistical details [file 508_2025_2508_MOESM1_ESM.docx]

**Supplemental material**

**A comparison of breast cancer incidence and cancer stages before and after the introduction of the Austrian national breast cancer screening program in the federal state of Salzburg: real-world data from the Tumor Registry Salzburg.**

**Ein Vergleich von Brustkrebsinzidenz und Tumorstadien vor und nach Einführung des nationalen Brustkrebsfrüherkennungsprogramms in Österreich im Bundesland Salzburg: Real-world Daten aus dem Salzburger Tumorregister.**

Simon Peter Gampenrieder^1,2^, Marc Vaisband^1,3^, Gabriel Rinnerthaler^1,2,4^, Lukas Weiss^1,2,6^, Bernhard Jaud^1,6^, Martin Sprenger^5^, Richard Greil^1,2,6^

**Running header:** Influence of the Austrian national breast cancer screening on breast cancer stage

*^1^ Department of Internal Medicine III with Haematology, Medical Oncology, Haemostaseology, Infectiology and Rheumatology, Oncologic Center, Salzburg Cancer Research Institute - Laboratory for Immunological and Molecular Cancer Research (SCRI-LIMCR), Paracelsus Medical University Salzburg, Salzburg, Austria;*

*^2^ Cancer Cluster Salzburg, Salzburg, Austria;*

*^3^ University of Bonn, Bonn, Germany;*

*^4^ Division of Oncology, Department of Internal Medicine, Medical University of Graz, Austria;*

*^5^ Institute of Social Medicine and Epidemiology, Medical University of Graz, Graz, Austria;*

*^6^ Tumor Registry Salzburg, Salzburg, Austria;*

**Appendix 1: Statistical details**

**1.1 Poisson rate parameter approximation**

For the equality testing of incidence rates in the Poisson model, we followed ([28](#_ENREF_28)) in calculated the age-standardized incidence rate as


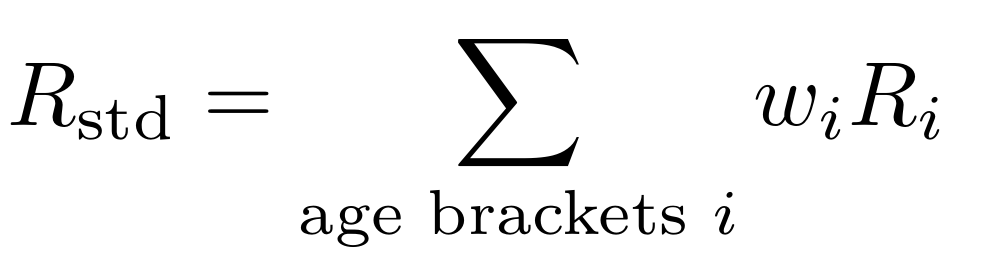


with the standard error estimate


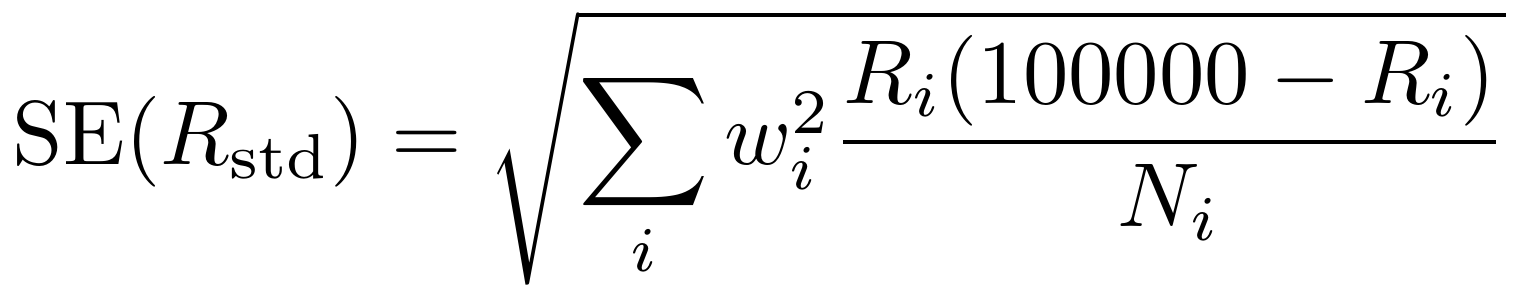


where the $w_{i}$ are the respective weights of the *i*-th age in the standard population, $N_{i}$are the raw case counts, and $R_{i}$ are the empirical incidence rates per 100,000 population for each age bracket. These rate estimates were then compared between the baseline and reference observation periods using the z-test, using the canonical test statistic:


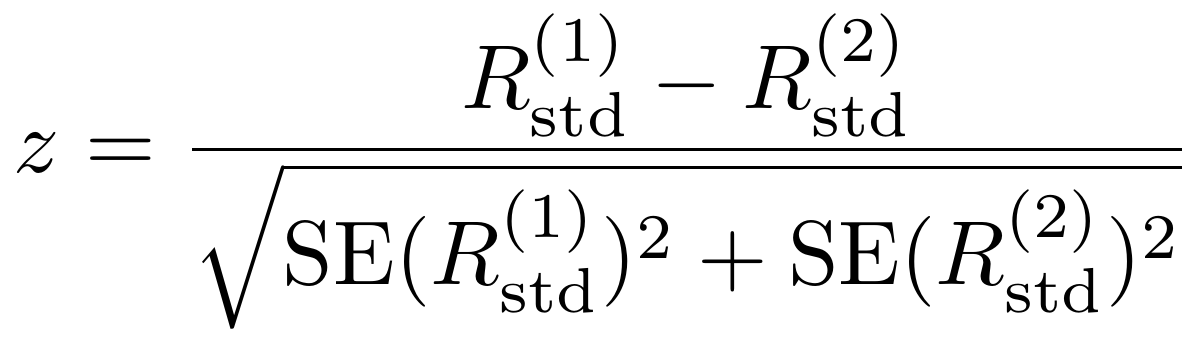


In order to estimate the power of this procedure, we additionallz calculated the minimal effect size which would have been necessary for a nominally significant test under equal standard error estimates. The results are reported in **Table S5** of the supplement.


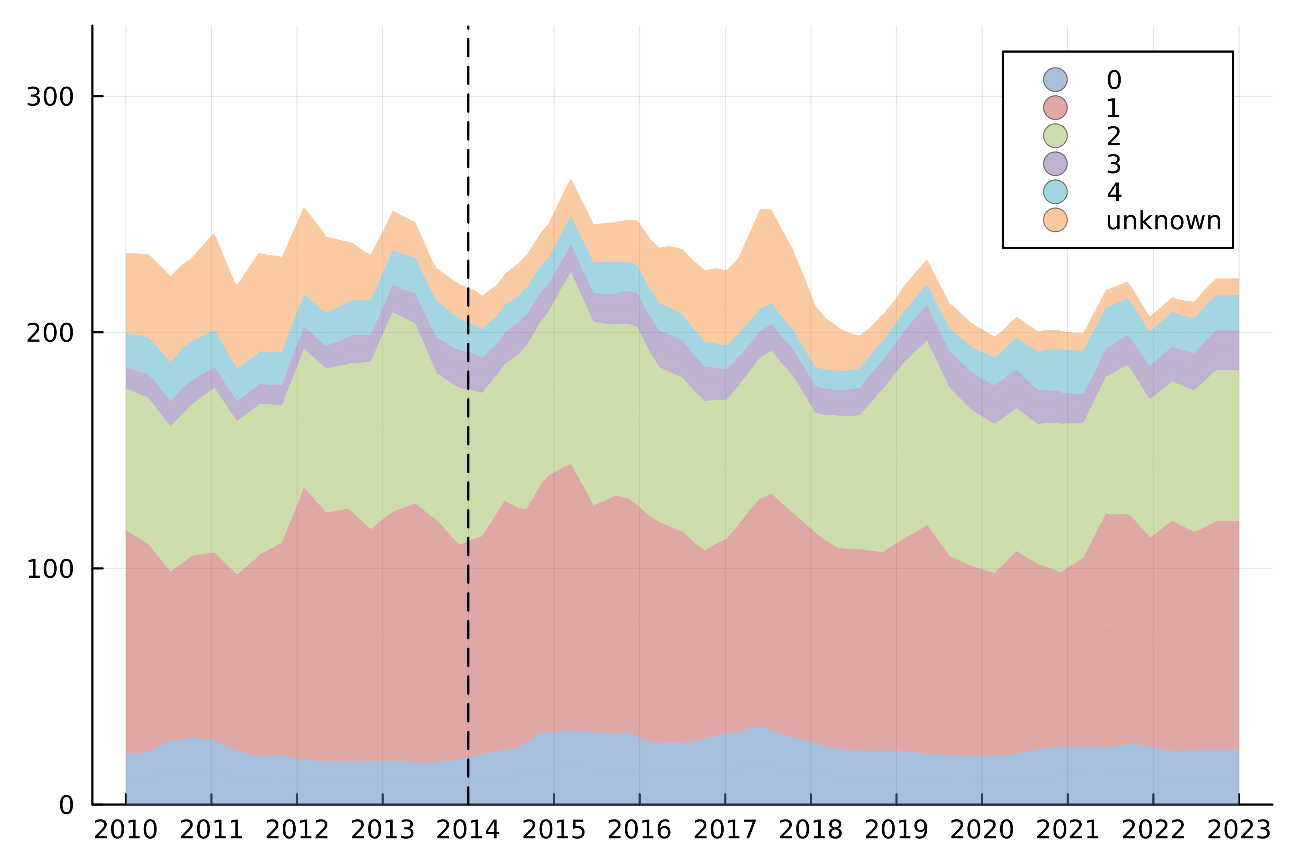


Figure S1. Graphical representation of the estimated breast cancer instantaneous incidence rate (per 100.000 women; not age-standardized) in the *Primary Study Population* (age 45-69; n = 2,310), obtained using non-parametric Bayesian intensity function estimation, over time in the federal state of Salzburg between 2010 and 2023, including the different stages at diagnosis.

Figure S2. Age-standardized incidence rate per year per 100,000 standard population (all stages) including the 95% confidence intervals in the *Primary Study Population* (age 45-69; n = 2,310).
* z-test comparing the incidence rates between 2010-2013 and 2016-2019.

Figure S3. Age-standardized incidence rate per year per 100,000 standard population including the 95% confidence intervals according to stage 0-I vs. II-IV in the *Primary Study Population* (age 45-69; n = 2,310).

Figure S4. Age-standardized incidence rate per year per 100,000 standard population according to stage 0-I vs. II-IV in the *Primary Study Population* (age 45-69; n = 2,310).

Figure S5. Age-standardized incidence rate per year per 100,000 standard population including the 95% confidence intervals according to nodal status (without stage IV) in the *Primary Study Population* (age 45-69; n = 2,310).

Figure S6. Percentage of breast cancer subtypes in the *Primary Study Population* (age 45-69; n = 2,310).

Table S1. Proportional distribution of the age-standardized incidence rate per year per 100,000 standard population according to stage in the *Primary Study Population.*

| **n = 2.310** | **2010-2022** | **2010-2013** | **2014-2015** | **2016-2019** | **2020-2022** | **p*** |
| --- | --- | --- | --- | --- | --- | --- |
| **Stage 0** | 12.6% | **12.2%** | 13.0% | **13.4%** | 11.6% | 0.380 |
| **Stage I** | 45.5% | **44.1%** | 47.9% | **46.2%** | 44.3% |  |
| **Stage II** | 28.3% | **28.2%** | 27.5% | **29.0%** | 28.0% |  |
| **Stage III** | 6.7% | **6.1%** | 6.1% | **6.9%** | 8.0% |  |
| **Stage IV** | 6.9% | **9.4%** | 5.5% | **4.5%** | 8.1% |  |
| **Stage 0-I** | 58.1% | **56.3%** | 60.9% | **59.6%** | 55.9% | 0.524 |
| **Stage II-IV** | 41.9% | **43.7%** | 39.1% | **40.4%** | 44.1% |  |
| **N0** | 69.5% | **64.8%** | 73.6% | **73.2%** | 66.7% | 0.538 |
| **N+** | 23.5% | **25.8%** | 20.6% | **22.2%** | 25.1% |  |

* Logistic regression including time period (2010-2013 vs. 2016-2019), age (45-54 vs. 55-64 and 45-54 vs. 65-74), interaction of age and time period and residence (rural vs. urban)

**Table S2.** Binary logistic regression for stage 0-I vs. II-IV in the Primary Study Population with diagnosis 2010-2013 or 2016-2019.

| **n = 1,364** | **Coefficient** | **95% CI** | **Standard deviation** | **z** | **p** |
| --- | --- | --- | --- | --- | --- |
| **Time period** 2010-2013* vs. 2016-2019 | -0.123 | -0.503 - 0.256 | 0.194 | -0.638 | **0.524** |
| **Age**  45-52* vs. 53-60 | 0.152 | -0.257 - 0.560 | 0.208 | 0.728 | **0.467** |
| 45-52* vs. 61-69 | -0.122 | -0.496 - 0.253 | 0.191 | -0.635 | **0.525** |
| **Interaction age / time period****  45-52* vs. 53-60 /  2010-2013* vs. 2016-2019 | -0.175 | -0.731 - 0.380 | 0.283 | -0.619 | **0.536** |
| 45-52* vs. 61-69 /  2010-2013* vs. 2016-2019 | 0.095 | -0.418 - 0.608 | 0.262 | 0.364 | **0.716** |
| **Residence** rural* vs. urban | -0.037 | -0.253 - 0.179 | 0.110 | -0.333 | **0.739** |

* reference

** included to correct for a potential change in age distribution between the different time periods

**Table S3.** Binary logistic regression for nodal status (N0 vs. N+) without stage IV in the *Primary Study Population* with diagnosis 2010-2013 or 2016-2019.

| **n = 1,277** | **Coefficient** | **95% CI** | **Standard deviation** | **z** | **p** |
| --- | --- | --- | --- | --- | --- |
| **Time period** 2010-2013* vs. 2016-2019 | -0.136 | -0.568 - 0.296 | 0.220 | -0.615 | **0.538** |
| **Age**  45-52* vs. 53-60 | 0.184 | -0.277 - 0.646 | 0.236 | 0.782 | **0.434** |
| 45-52* vs. 61-69 | -0.123 | -0.557 - 0.311 | 0.222 | -0.555 | **0.579** |
| **Interaction age / time period****  45-52* vs. 53-60 /  2010-2013* vs. 2016-2019 | -0.408 | -1.049 - 0.234 | 0.327 | -1.246 | **0.213** |
| 45-52* vs. 61-69 /  2010-2013* vs. 2016-2019 | -0.036 | -0.633 - 0.561 | 0.305 | -0.117 | **0.907** |
| **Residence** rural* vs. urban | -0.028 | -0.281 - 0.224 | 0.129 | -0.220 | **0.826** |

* reference

** included to correct for a potential change in the distribution between the different time periods

**Table S4.** Ordinal logistic regression for stage 0-IV in the *Primary Study Population* after data imputation (Mx 🡪 M0 if stage I or II).

| **n = 1,550** | **Coefficient** | **95% CI** | **Standard deviation** | **z** | **p** |
| --- | --- | --- | --- | --- | --- |
| **Time period** 2010-2013* vs. 2016-2019 | -0.146 | -0.468 - 0.176 | 0.164 | -0.888 | **0.374** |
| **Age**  45-52* vs. 53-60 | 0.068 | -0.273 - 0.409 | 0.174 | 0.392 | **0.695** |
| 45-52* vs. 61-69 | -0.015 | -0.322 - 0.293 | 0.157 | -0.095 | **0.925** |
| **Interaction age / time period****  45-52* vs. 53-60 /  2010-2013* vs. 2016-2019 | 0.003 | -0.473 - 0.479 | 0.243 | 0.012 | **0.991** |
| 45-52* vs. 61-69 /  2010-2013* vs. 2016-2019 | 0.004 | -0.432 - 0.441 | 0.223 | 0.019 | **0.985** |
| **Residence** rural* vs. urban | -0.024 | -0.209 - 0.161 | 0.094 | -0.252 | **0.801** |

* reference

** included to correct for a potential change in the distribution between the different time periods

Table S5 Calculation of the minimum difference size for nominally significant test for a ceteris-paribus comparison of age-standardized incidence rates between 2010-2013 and 2016-2019.

|  | **2010-2013** | 95%CI | **2016-2019** | 95%CI | p | **Difference in incidence rate**  **2010-2013 vs. 2016-2019** | **Minimum difference size for nominally significant test with unchanged standard errors** |
| --- | --- | --- | --- | --- | --- | --- | --- |
| **All stages*** | **176.0** | 149.3-206.0 | **193.9** | 167.0-224.0 | **0.371** | **17.9** | **39.4** |
| **Stage 0** | **21.5** | 12.9-33.6 | **26.1** | 16.8-38.5 | **0.525** | **4.6** | **14.1** |
| **Stage I** | **77.6** | 60.3-98.4 | **89.6** | 71.5-110.7 | **0.376** | **12.0** | **26.5** |
| **Stage II** | **49.6** | 35.8-66.2 | **56.2** | 41.9-73.1 | **0.539** | **6.6** | **21.0** |
| **Stage III** | **10.8** | 5.0-20.2 | **13.5** | 7.2-23.1 | **0.602** | **2.7** | **10.1** |
| **Stage IV** | **16.5** | 8.9-27.2 | **8.7** | 3.6-16.6 | **0.141** | **7.8** | **10.4** |
| **N0** | **114.1** | 92.9-138.7 | **142.0** | 118.8-167.7 | **0.095** | **27.9** | **32.7** |
| **N+** | **45.4** | 32.2-61.5 | **43.0** | 30.6-30.6 | **0.807** | **2.4** | **19.2** |
| **Nodal status unknown** | **0.0** | 0.0-0.0 | **0.3** | -0.82-1.39 | **0.617** | **0.3** | **1.1** |

* Stages according to AJCC version 8
